# Breastfeeding and the incidence of endometrial cancer: A meta-analysis

**DOI:** 10.18632/oncotarget.5049

**Published:** 2015-09-05

**Authors:** Baojian Zhan, Xiaoqin Liu, Fang Li, Dongfeng Zhang

**Affiliations:** ^1^ Department of Epidemiology and Health Statistics, the Medical College of Qingdao University, Qingdao, Shandong Province, People's Republic of China

**Keywords:** endometrial cancer, breastfeeding, meta-analysis, dose–response

## Abstract

Several epidemiological studies have investigated the association between breastfeeding and endometrial cancer (EC). However, the results of the studies are controversial. Thus, we conduct this meta-analysis to explore the association between breastfeeding and EC and to evaluate the possible does-response relationship between duration of breastfeeding and EC. PubMed, Web of Science, Chinese National Knowledge Infrastructure, China biology medical literature database, Wan fang databases and Database of Chinese Scientific and Technical Periodicals were searched for eligible observational studies up to 11 July 2015. Random effects model was used to calculate the pooled relative risks (*RR*s) and restricted cubic spline model was adopted for the does-response analysis.

Fifteen articles with 623570 participants were identified. The *RR*s of these studies suggested that breastfeeding was associated with the reduced risk of EC (high versus low/no: *RR* = 0.74; 95% confidence interval (*CI*), 0.58–0.95). In subgroup analyses, a significant association of breastfeeding with EC risk was found in Asia (*RR* = 0.57, 95% *CI* 0.37–0.87), and an inverse association of breastfeeding with EC risk was found in cohort studies (*RR* = 0.62, 95% *CI* 0.41–0.94). The results were also significant after adjusted for hormone use (*RR* = 0.63, 95% *CI* 0.41–0.97) and body mass index (*RR*=0.65, 95% *CI* 0.44–0.96). A linear relationship was found of breastfeeding with EC (*p* for nonlinearity = 0.93), and it indicated that EC risk decreased by 1.2% for one month increment of breastfeeding. This meta-analysis indicates that long term breastfeeding might be associated with decreased risk of EC.

## INTRODUCTION

Endometrial cancer (EC) is one of the most common malignancies among women. There were 320,000 new cases diagnosed in 2012 worldwide [1, 2], and the numbers of new EC cases and deaths were on the increase [1, 3, 4]. Present studies indicate that genetic factors, anthropometric factors and life style factors may be related to EC risk [5–7]. EC is thought to be caused by the continuous stimulation of estrogen [8], so conditions related to estrogen may alter EC risk, such as menstrual history, parity and exogenous hormones [9–11].

Breastfeeding is an essential biological function of humans and the beneficial effects of breastfeeding for both mother and child are widely acknowledged [12–14]. Meta-analyses found that breastfeeding was associated with breast cancer, ovarian cancer and childhood leukemia [15–17]. Breastfeeding may influence the risk of EC, because of the hormonal changes during breastfeeding.

Several epidemiological studies have investigated the association between breastfeeding and EC [18–22]. However, the results of the studies are inconsistent. Some studies suggest that breastfeeding can reduce the risk of endometrial cancer [21–23], while Herrinton LJ, and Dossus, L found no association between breastfeeding and EC [19, 24]. We conduct this meta-analysis to explore the association between breastfeeding and EC and to evaluate the possible does-response relationship of duration of breastfeeding with EC.

## RESULTS

### Literature search and study characteristics

**Figure 1:**
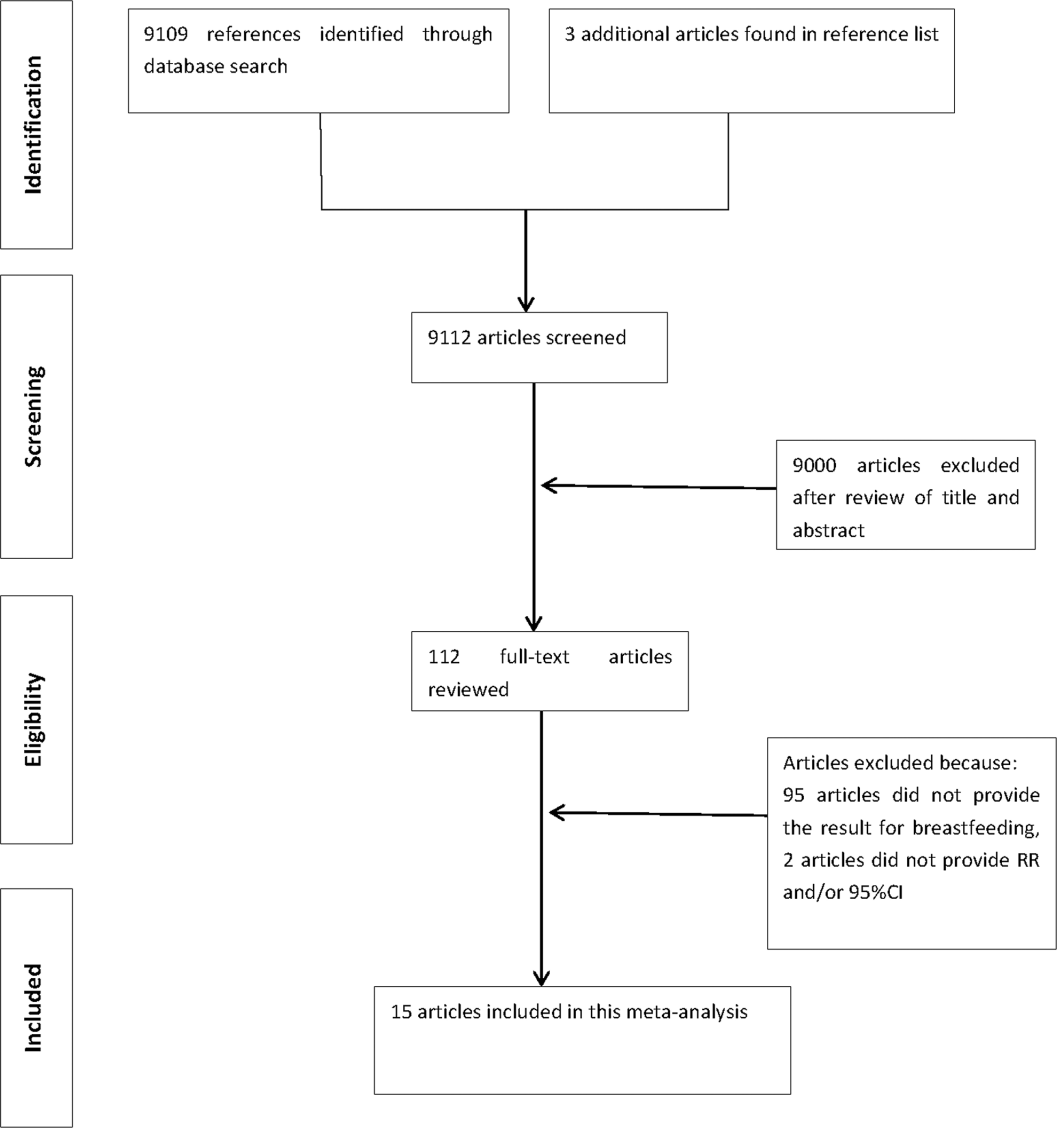
Flowchart of the selection of studies included in the meta-analysis

We identified 9109 articles by the search strategy and 3 articles by searching reference lists, of which 9000 articles were excluded after review of the title/abstract or which were duplicated (Figure 1). One hundred and twelve full-text articles were reviewed. We further excluded 97 articles that did not provide *RR*s concerning the relation between breastfeeding and EC. Eventually 15 published articles [18–32] with 623570 participants were included in this analysis, of which 8 studies [19, 21–23, 25–27, 30] provided different duration of breastfeeding for the does-response relationship. The detailed characteristics of the 15 studies are shown in Table 1.

**Table 1:** Characteristics of studies included in the meta-analysis of breastfeeding and endometrial cancer

Author(year)	Country	Study design, age	Sample size (cases)	RR (95%CI) for highest vs. lowest category	Adjustment for covariates
Sugawara et al.(2013)	Japan	cohort,40–79	19,848(32)	0.31(0.12–0.81)	Age, BMI, family history of cancer, education, job status, smoking status, alcohol consumption, time spent walking, total calorie intake, menopausal status, age at menarche, age at first delivery, number of deliveries, history of oral contraceptive drug use, history of hormone replacement therapy
Dossus et al.(2009)	Denmark, France, Germany, Greece, Italy, the Netherlands, Norway, Spain, Sweden and United Kingdom	cohort,-	302,618(1017)	0.77(0.54–1.11)	Age, BMI, physical activity, alcohol, diabetes, smoking status, education
Zucchetto et al.(2009)	Italy	cc,60/61	1,362(454)	1.33(0.95–1.85)	Age, period of interview, BMI, age at menarche, age at menopause, parity, oral contraceptive use, when appropriate
Brinton et al.(2007)	Polish	cc,20–74	2,476(551)	0.72(0.4–1.2)	Age, study site, years of education, age at menarche, number of full-term births, ever use of oral contraceptives, ever use of oral hormones, ever smoking, BMI
Okamura et al.(2006)	Japan	cc,51.6/49.6	251(155)	0.37(0.17–0.82)	Age, BMI, oral contraceptive use
Wernli et al.(2006)	China	cohort,-	259,640(206)	0.62(0.35–1.09)	Age at baseline, number of live births
Xu et al.(2004)	China	cc,30–69	1,559(754)	0.54(0.33–0.87)	Age, BMI, family history of cancer, number of pregnancies, history of abortion, duration of menstruation(Continued )
Herrinton et al.(2001)	United States	cc,20–54	896(179)	0.95(0.65–1.4)	
Newcomb et al.(2000)	United States	cc,40–79	2,239(586)	0.84(0.52–1.4)	Age, smoking status, education, body mass, postmenopausal hormone therapy, parity
Salazar-Martinez et al.(1999)	Mexico	cc,57.1/54.6	837(85)	0.33(0.17–0.65)	Age, hormonal use, number of pregnancies, smoking, diabetes mellitus, hypertension, physical activity, menopausal status, BMI
Hirose et al.(1999)	Japan	cc,48.5/56.6	26,953(1465)	1.48(0.63–3.49)	Age, BMI
Rosenblatt et al.(1995)	Australia Israel Chile, China, Philippines, and Thailand	cc,-	1,069(136)	0.23(0.08–0.68)	Number of pregnancies, age at menarche
Brinton et al.(1992)	United States	cc,-	702(405)	1.01(0.6–1.6)	Age, number of births, years of education, recent weight, oral contraceptive use, menopausal estrogen use
Cusimano et al. (1989)	Italy	cc,61.7/60.2	480(57)	2.94(0.68–12.5)	Unclear
Elwood et al.(1977)	United States	cc,55–59	2,640(622)	1(0.7–1.5)	Age, marital status, parity, age at first birth, age at menarche, age at natural menopause, history of stillbirth or miscarriage

Among the 15 studies, five studies [18, 20, 24, 26, 30] were conducted in North America, five studies [23, 27, 28, 30, 31] were conducted in Asia, and four studies [19, 25, 29, 32] were conducted in Europe. Regarding to the study type, 12 studies [18, 20–26, 29–32] were case-control (CC) designs and three [19, 27, 28] were cohort studies. The Newcastle-Ottawa score of quality assessment showed that the scores of 13 studies were more than 7, indicating that the methodological quality was generally good (Supplementary Table S1).

### Quantitative synthesis

#### Overall association between breastfeeding and EC risk

**Figure 2:**
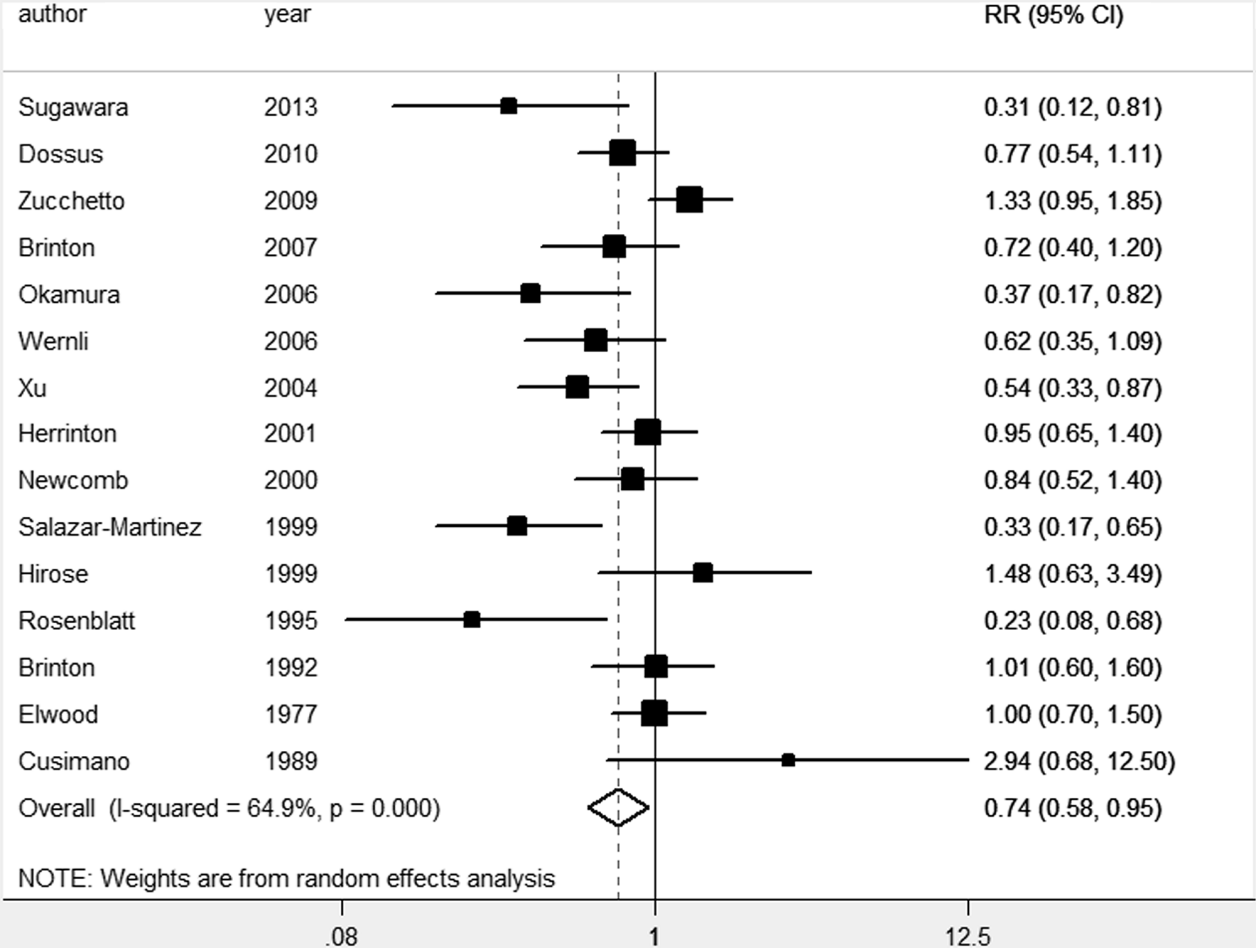
Forest plot for the pooled relative risk of breastfeeding for EC

**Figure 3:**
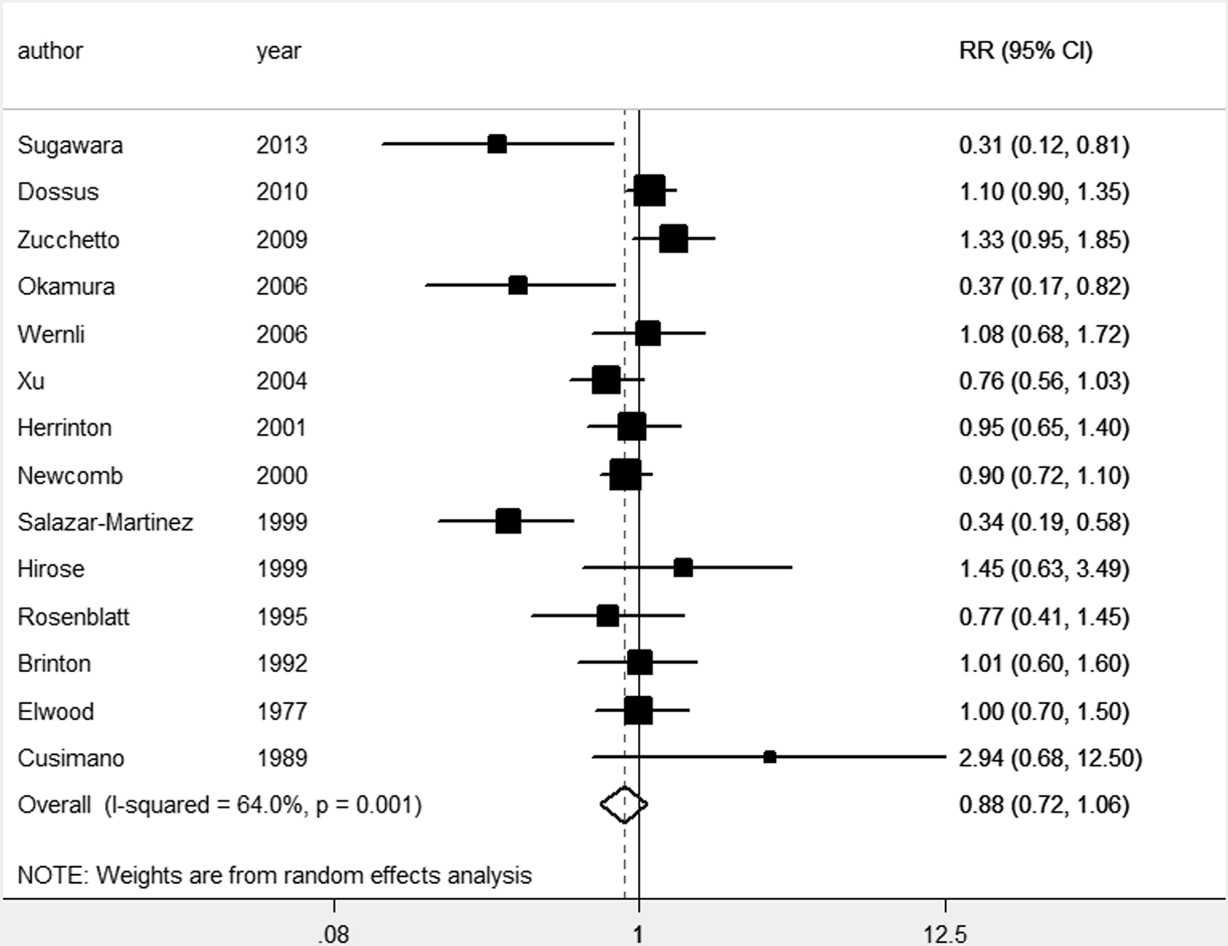
Forest plot for the pooled relative risk of breastfeeding for EC (the ever breastfeeding compared with the reference group)

The pooled *RR* of overall data by the random effects model (REM) was 0.74 (95% *CI* 0.58–0.95). We have separated the results by ever breastfeeding and the duration of breastfeeding. The pooled *RR* of 14 studies [18–24, 26–32] was 0.88 (95% *CI* 0.72–1.06) for the ever breastfeeding compared with the reference group. The association between breastfeeding and risk of EC is provided in Figure 2 and Figure 3.

#### Subgroup analysis by continent

We found a strong and significant association of breastfeeding with EC risk in Asia (*RR* = 0.57, 95% *CI* 0.37–0.87), but the association was not significant in Europe (*RR* = 1.00, 95% *CI* 0.66–1.53) and in North-America (*RR* = 0.82, 95% *CI* 0.60–1.13).

#### Subgroup analysis by study design

Inverse association was found for cohort studies. The pooled *RR* for cohort studies was 0.62 (95% *CI* 0.41–0.94; *I^2^* = 36.5%; *p* > 0.05), and the pooled *RR* for case-control studies was 0.78 (95% *CI* 0.59–1.04; *I^2^* = 67.9%; *p* < 0.05).

#### Subgroup analysis by adjustment for covariates

We found no significant association in stratified analysis by adjustment (yes or no) for the following covariates: postmenopausal hormone replacement therapy use, oral contraceptive use, body mass index (BMI), menarche age and menopausal status. Moderate or lower between-study heterogeneity was found in all analyses. Table 2 showed the results from all analyses.

**Table 2:** Pooled measures on the relation of breastfeeding and EC

			Heterogeneity		
Subgroup	Number of studies	RR (95%CI)	I^2^ (%)	*P* value	Article included
All	15	0.74(0.58 to 0.95)	64.9	0.00	18–32
Ever/never	14	0.88(0.72 to 1.06)	64.0	0.00	18–31
Study region					
Asia	5	0.57 (0.37 to 0.87)	47.6	0.11	23,27,28,30,31
Europe	4	1.00 (0.66 to 1.53)	63.9	0.04	19,26,29,32
North America	5	0.82 (0.60 to 1.13)	56.1	0.06	18,20,22,24,25,
Other	1	0.23 (0.08 to 0.66)	-	-	21
Study design					
Cohort study	3	0.62(0.41 to 0.94)	36.5	0.21	19,27,28
Case-control study	12	0.78(0.59 to 1.04)	67.9	0.00	18,20–26,29–32
Adjustment for hormone use					
Yes	5	0.63(0.41 to 0.97)	61.1	0.04	18,22,25,26,28
No	9	0.77(0.58 to 1.03)	67.4	0.00	19–21,23, 24,27,29–31
Adjustment for oral contraceptive use					
Yes	5	0.72(0.44 to 1.19)	74.6	0.00	18,26,28,29,31
No	9	0.71(0.54 to 0.93)	58.0	0.02	19–25,27,30
Adjustment for body mass index					
Yes	8	0.65(0.44 to 0.96)	74.7	0.00	19,22,23, 26,28–31
No	7	0.83(0.63 to 1.08)	41.4	0.13	18,20,21, 24,25,27
Adjustment for menarche age					
Yes	5	0.70(0.42 to 1.17)	76.7	0.00	20,21,26,28,29
No	9	0.71(0.55 to 0.92)	51.6	0.04	18,19,22–25,27,30,31
Adjustment for menopausal status					
Yes	5	0.65(0.38 to 1.11)	82.6	0.00	20,22,23,28,29
No	9	0.76(0.60 to 0.97)	41.2	0.09	18,19,21, 24–27,30,31

### Dose–response analysis

**Figure 4:**
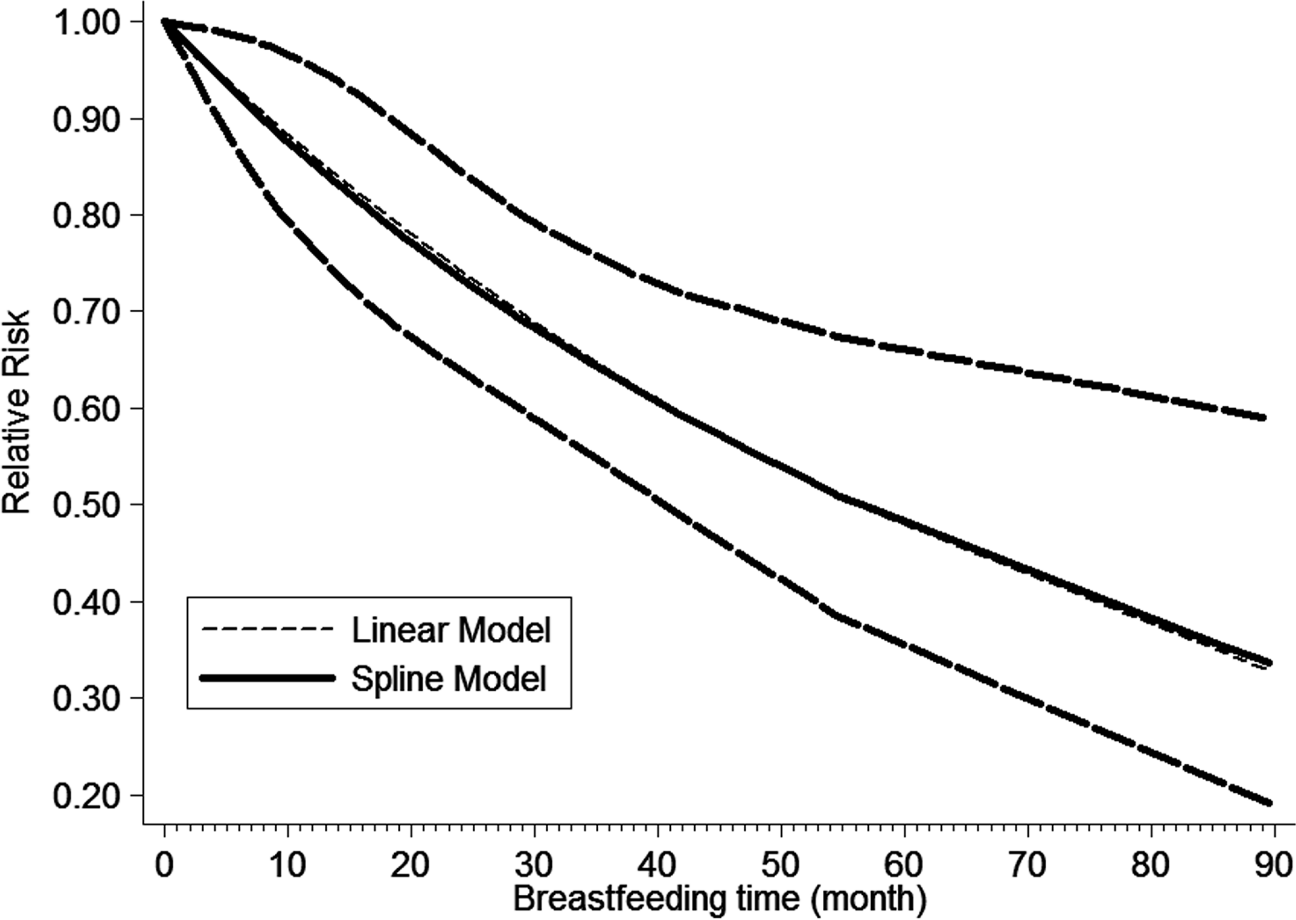
The dose–response analysis between breastfeeding and EC risk with restricted cubic splines in a multivariate random-effects dose–response model The solid line and the long dash line represent the estimated relative risk and its 95% confidence interval. Short dash line represents the linear relationship

For dose–response analysis, eight studies including 2919 EC cases [19, 21–23, 25–27, 30] provided the data. We found a linear relationship of breastfeeding with EC (*p* for nonlinearity = 0.93). Compared with 0 month, the *RR*s (95% *CI*) of EC were 0.99 (0.99–1.00), 0.92 (0.86–0.98), 0.83(0.74–0.94) and 0.79 (0.69–0.90) for 0.5, 6, 14 and 18 months separately. The dose–response analysis suggested that EC risk decreased by 1.2% for one month increment of breastfeeding (Figure 4).

### Sources of heterogeneity and sensitive analysis

Moderate between-study heterogeneity was found in this analysis. Exploratory univariate meta-regression was performed with the covariates of published year, study area, study type, cases and sample size. However, results showed that no covariate had a significant impact on between-study heterogeneity (published year: *p* = 0.354, study region: *p* = 0.879, study type: *p* = 0.441, cases: *p* = 0.143, sample size: *p* = 0.911). The ‘leave one-out’ sensitive analysis [33] was carried out to assess the key studies contributed to this high between-study heterogeneity. After excluding three articles conducted by Rosenblatt KA et al [21], Salazar-Martinez E [22] and Zucchetto A [29], no significant heterogeneity (*I^2^* = 42.1%, *p* > 0.05) was found, and the pooled *RR* was 0.78 (95% *CI* 0.64–0.96).

### Influence analysis

No individual study was found to have excessive influence on the pooled effects for conclusion (specific data were not provided).

### Publication bias evaluation

**Figure 5:**
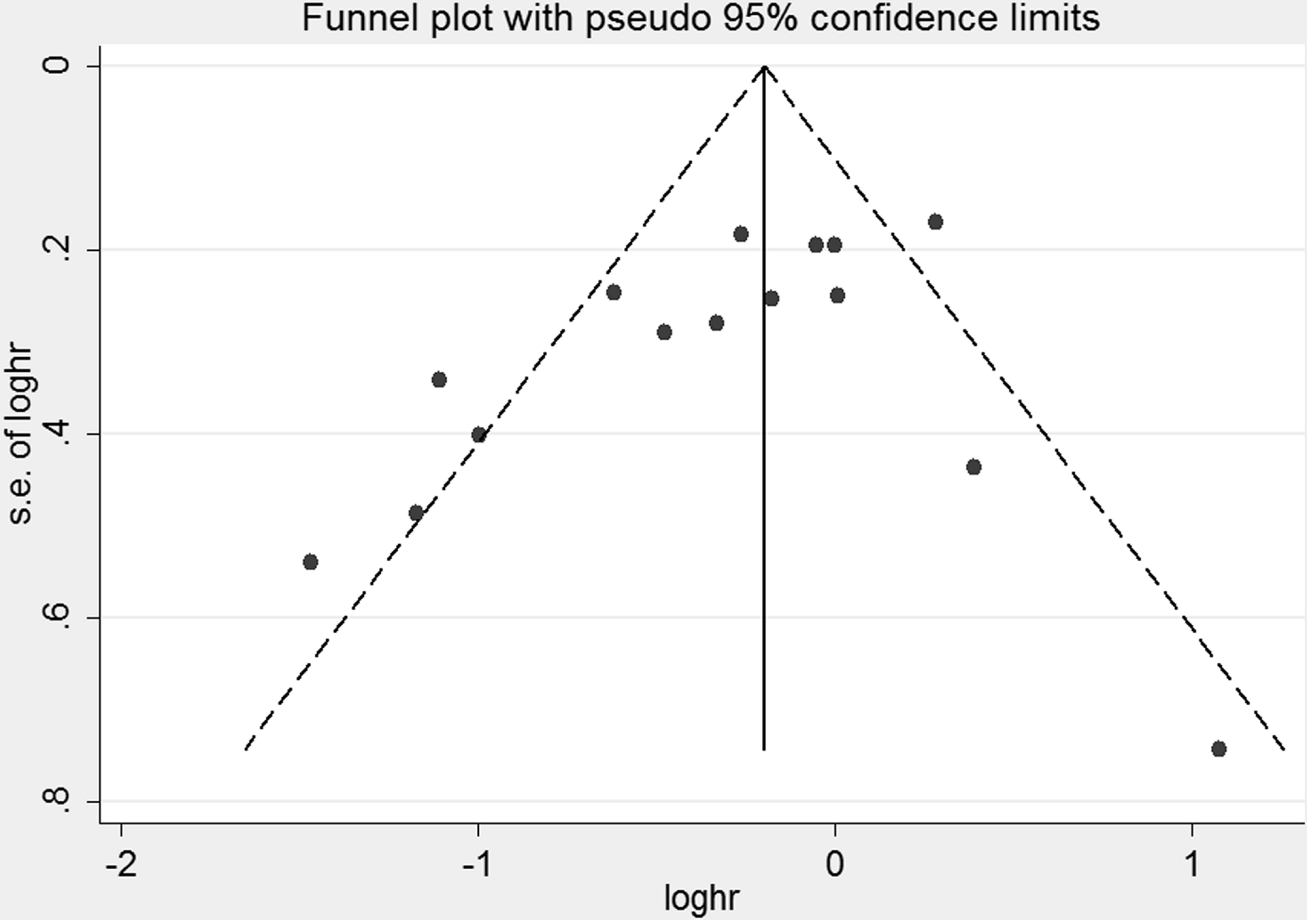
Funnel plot for the analysis of breastfeeding and EC risk after removing three studies that had a strong effect on heterogeneity

Publication bias was detected by Egger test and the visual inspection of the funnel plot. No significant asymmetry of the funnel plot was found in the Egger test (*p* = 0.097) (Figure 5).

## DISCUSSION

To our knowledge, this is the first meta-analysis to examine the association between breastfeeding and EC. From the results we found that breastfeeding was associated with a statistically significant decreased risk of EC, and there was a notable linear relationship between duration of breastfeeding and risk of EC. Dose–response analysis revealed that EC risk decreased by 1.2% for 1 month increment of breastfeeding. The summary *RR*s (95% *CI*s) of EC did not substantially change in the sensitivity analysis. Subgroup analysis by study design showed the inverse association between breastfeeding and EC for cohort studies. These results proved that our results were stable and credible.

We found a strong and significant association of breastfeeding with EC risk in Asia (*RR*=0.57, 95% *CI* 0.37–0.87). In Asia, Europe and North America, the traditional culture and people's lifestyles are different. First, more Asians tend to choose breastfeeding as the way to feed children with respect to the Europeans and North Americans, and the duration of breastfeeding is longer [31]. Second, genetic factors are various among different continents. Third, the BMI of Europeans and North Americans is higher than Asians, which has been associated with a higher risk of EC [34]. These may explain the different results of the three continents.

Several hypotheses have been provided to describe the association between breastfeeding and EC. In general, EC is related to female hormones [35–37]. When endometrium is continuously stimulated by estrogens, mitotic activity is promoted. The increasing possibility of genetic mutations results in a carcinoma eventually [38, 39]. During lactation, pituitary and ovarian hormones are suppressed [40, 41], reducing stimulation of the endometrium by estrogen [42]. Another hypothesis that has been raised to explain endometrial carcinogenesis is the “unopposed estrogen” hypothesis [43]. It asserts that a high level of estrogens stimulates cells proliferation of the endometrium, when they are not counterbalanced by progesterone [42]. During breastfeeding the decline of estrogen is more significant than that of progesterone [40], so estrogen is opposed by progesterone. Overall, breastfeeding may alter EC risk through the hormonal [44]. On the other hand, longer duration of breastfeeding could strongly decrease the risk of overweight [45], which could subsequently and substantially increase the risk of endometrial cancer [46].

Between-study heterogeneity is common in meta-analysis [47], and it is demanded to explore the potential sources of between-study heterogeneity. Our meta-analysis showed moderate between-study heterogeneity. The different characteristics of each study may result in heterogeneity. In our meta-analysis, the number of the studies in our analysis is limited. Besides, the adjustment factors are various among the studies. For example, Cusimano et al. [32] did not indicate the adjustment factors in his research. On the other hand, Rosenblatt et al. [21] used extreme category greater than 72 months as the highest category, while Hirose et al. [30] used extreme category more than 12 months as the highest category. We used meta-regression to explore the potentially important causes of the between-study heterogeneity. However, our meta-analysis did not find the covariates as the contributors to the between-study heterogeneity. After the ‘leave one-out’ sensitive analysis, no significant heterogeneity (*I^2^* = 42.1%, *p* > 0.05) was found. The studies that we excluded in the ‘leave one-out’ sensitive analysis contained a relatively small number of cases, and the adjustment factors in the study of Rosenblatt KA et al [21] was less than others. Furthermore, the pooled *RR* was 0.78 (95% *CI* 0.64–0.96) after the ‘leave one-out’ analysis.

A major strength of this meta-analysis is the large number of included participants, allowing us to get the stable results. Second, most included studies had adjusted for potential confounders, increasing the credibility of the results. Third, dose–response analysis was performed to better describe the association of breastfeeding time with EC risk. In addition, the subgroup analysis by study design strongly identified the effect of breastfeeding on EC, the pooled *RR* of cohort studies was 0.62 (95% *CI* 0.41–0.94).

However, the limitations of our study should also be considered. First, further adjustments need to be conducted to clarify the independent role of breastfeeding to EC. Second, the adjustment factors of each study were different. Third, majority studies of this analysis were case-control design. Considering the recall bias of case-control studies, the effect of breastfeeding on EC requires confirmation in large cohort studies.

In summary, this meta-analysis confirms an inverse association between breastfeeding and EC risk especially in Asia. Our findings also confirm that longer time of breastfeeding may reduce the risk of EC. The association of breastfeeding and EC deserves further investigation.

## MATERIALS AND METHODS

### Literature search strategy

The initially relevant studies from PubMed, Web of Science, Chinese National Knowledge Infrastructure, China biology medical literature database, Wan fang databases and Database of Chinese Scientific and Technical Periodicals were identified up to 11 July 2015. All the articles were restricted to English or Chinese language. The following strategy was used in this search: ((((((reproductive) OR reproduction))) OR ((breastfeeding) OR lactation))) AND ((endometrial cancer OR endometrial neoplasm OR endometrial carcinoma OR uterine corpus)). In addition, the reference lists from retrieved articles were also reviewed to identify relevant studies [34, 46].

### Inclusion criteria

All studies were reviewed independently by the first and the second authors. If the two authors disagreed about the eligibility of an article, it was resolved by consensus with the third author. The studies must meet the following inclusion criteria: (1) case-control or cohort studies published as original studies to evaluate the association between breastfeeding and risk of EC incidence. (2) Odds ratio (OR), relative risk (*RR*) or hazard ratio with 95% confidence interval (*CI*) were available. (3) The number of cases and participants or person-years for different duration of breastfeeding must be provided in order to do the dose–response analysis. If multiple articles were published from the same study, we choose the complete one or the study which can provide sufficient detail of data.

### Data extraction

The following items were extracted from each study: the first author's name, sample size, EC cases, study type, publication year, continent, follow-up years for cohort studies, adjustment for potential confounding, *RR*s and 95% *CI*s (we presented all results with *RR* for simplicity). We extracted *RR*s adjusted with the most confounders. For dose–response analysis, the number of cases, participants (person-years), and *RR* (95% *CI*) for each category of breastfeeding were extracted, as well as the different duration of breastfeeding [48]. The study quality was assessed using the Newcastle-Ottawa quality assessment scale.

### Statistical analysis

Pooled measure was calculated as the inverse variance-weighted method of the logarithm of *RR* with 95% *CI* by the inverse variance-weighted method. We used the Q test and the *I^2^* statistic to assess the heterogeneity among studies [49]. The REM was applied as the pooling method [50].

Meta regression and subgroup analyses were conducted to explore the possible sources of between-study heterogeneity. The restricted maximum likelihood method (REML) was used to estimate the additive (between-study) component of variance tau^2 in meta-regression. The ‘leave one-out’ sensitive analysis [33] was carried out to evaluate the key studies. Influence analysis was performed to validate the stability of outcomes by removing one study at a time. Publication bias was assessed by visual inspection of the funnel plot and Egger's test [51].

For the does-response analysis, the method described by Orsini et al [52] was used. A two-stage random-effects dose–response meta-analysis was performed to compute the trend from the correlated log *RR* estimates across duration of breastfeeding. First, we created a restricted cubic spline model with three knots at the 10 th, 50 th, and 90 th centiles. Then the REML method was used to combine the study-specific estimates in a multivariate random-effects meta-analysis [53]. By testing the null hypothesis that the coefficient of the second spline is equal to 0, *p* value for nonlinearity was calculated. All statistical analyses were conducted by Stata V.12.0 (Stata Corp, College Station, Texas, USA). A 2-sided and a *p* < 0.05 was considered statistically significant.

## SUPPLEMENTARY TABLE


