# Supplementary material for: Breastfeeding and the incidence of endometrial cancer: A meta-analysis
Source: Oncotarget. 2015 Sep 5;6(35):38398–409. doi: 10.18632/oncotarget.5049 (PMC4742008; doi:10.18632/oncotarget.5049)
Supplement: Supplementary file 1 [file oncotarget-06-38398-s001.pdf]

SUPPLEMENTARY TABLE

Supplementary Table S1: Quality assessment of studies included in the meta-analysis

author	study design	selection	comparability	Outcome/exposure	Overall quality
Sugawara	cohort study	***	**	***	8
Dossus	cohort study	***	**	**	7
Wernli	cohort study	***	**	***	8
Cusimano	case-control study	****	*	*	6
Zucchetto	case-control study	***	**	**	7
Rosenblatt	case-control study	***	**	**	7
Brinton	case-control study	****	*	**	7
Newcomb	case-control study	****	*	**	7
Okamura	case-control study	***	*	**	6
Salazar-Martinez	case-control study	***	**	**	7
Herrinton	case-control study	****	**	**	8
Xu	case-control study	****	*	**	7
Brinton	case-control study	***	**	**	7
Elwood	case-control study	****	**	*	7
Hirose	case-control study	***	**	**	7
